# Markers of long term silent carriers of *Streptococcus equi* ssp. *equi* in horses

**DOI:** 10.1111/jvim.15939

**Published:** 2020-10-19

**Authors:** John Pringle, Monica Venner, Lisa Tscheschlok, Andrew S. Waller, Miia Riihimäki

**Affiliations:** ^1^ Department of Clinical Sciences Swedish University of Agricultural Sciences Uppsala Sweden; ^2^ Equine Veterinary Clinic Destedt Germany; ^3^ Animal Health Trust Suffolk UK

**Keywords:** antigen A, antigen C. silent carrier, equine, iELISA, infection

## Abstract

**Background:**

Difficulty in detection of silent carriers of *Streptococcus equi* is a key reason for its continued spread to immunologically naïve groups of horses.

**Objective:**

To determine whether clinical examination, markers of inflammation, or serology differentiate silent carriers of *S. equi* in recovered comingled horses.

**Animals:**

Ninety‐eight warmblood yearlings and 72 unaffected mares on a large breeding farm (outbreak A), 38 mature Icelandic horses at a riding stable (outbreak B), and 27 mixed breed horses at a boarding stable (outbreak C).

**Methods:**

Prospective observational study 6 months to 2 years after strangles outbreaks. Carriers were defined as any animal positive on culture or qPCR to *S. equi* from nasopharyngeal lavage or guttural pouch endoscopy and lavage. Most horses had complete physical exams and 1 group included evaluation of white blood cell counts and serum amyloid A. Sera from all horses was tested for antibodies to antigens A and C of *S. equi* using an enhanced indirect ELISA. Descriptive statistics were calculated. Data were compared using paired *t* tests, Wilcoxon ranked test, chi square, or the Fishers exact test. Significance was set at *P* < .05.

**Results:**

Apart from weanlings at 6 months in outbreak A, there was no significant association between any clinical markers or serology with carrier state (*P* = .06‐1). Moreover, 3/12 culture positive carriers were seronegative to *S. equi*.

**Conclusions and Clinical Importance:**

Silent carriers of *S. equi* do not differ clinically or on markers of inflammation to their noncarrier herd‐mates. Moreover, serology alone will not distinguish carriers in comingled horses.

**Abbreviations**
_ΔΔ_CT: log fold changes on qPCR
GPL: guttural pouch lavage
iELISA: indirect enzyme linked immunosorbent assay
NPL: nasopharyngeal lavage
qPCR: real time quantitative PCR
SAA: serum amyloid A
WBC: white blood cell count

## 1 INTRODUCTION

*Streptococcus equi* subspecies *equi* (*S. equi*) causes the serious and highly contagious respiratory disease strangles in horses over most of the world.^1^, ^2^ Recognition of the clinically silent carrier state,^3^ as predicted at the turn of the last century,^4^ pointed to key reasons for persistence of this disease in the horse population, and spread to immunologically naïve groups of horses.

Differentiation of these silent long‐term carriers from noncarrier herd‐mates after an outbreak is essential for targeted management. Both directed treatment of carriers and biosecurity measures minimize the risk such animals pose in triggering strangles outbreaks in new groups of horses. Guidelines for detection of *S. equi* in carriers, either by culture or detection of bacterial DNA by qPCR, have been described^2^ consisting of either nasopharyngeal washes and/ or guttural pouch endoscopy and lavage.^5^ Ideally, in unvaccinated populations incorporation of the recently developed combined iELISA to identify animals that have been exposed to *S. equi*, followed with targeted testing by qPCR for those animals which may still harbor *S. equi* is advised to optimize within herd biosecurity measures.^2^ However, for many countries such early intervention is not applied widely, with uniform diagnostic testing lacking in most strangles outbreaks.^6^ Moreover, apart from earlier preliminary studies involving an iELISA that targeted the SeM allele 1,^7^ the clinical and serological phenotype using the more recently developed enhanced iELISA for detection of silent carriers of strangles in comparison to noncarrier herd‐mates remains to be investigated. It would thus be of value to determine whether, after more than several months of unassisted recovery from a strangles outbreak, silent carriers of *S. equi* can be readily differentiated from comingled noncarrier stable mates, based on clinical examination, guttural pouch endoscopy, blood sample analysis for markers of inflammation or serology.

We examined the occurrence of long‐term carriers of *S. equi* prospectively in 3 strangles outbreaks with differing management regimens; a large breeding farm with recurrent outbreaks of strangles, a closed riding stable with mature horses only, and a boarding stable for convalescent horses of varying breeds and ages. The aims were to determine whether horses identified as persistent carriers of *S. equi* following natural recovery from a strangles outbreak differed from noncarrier comingled herd‐mates, based on clinical or selected laboratory analyses.

## 2 MATERIALS AND METHODS

Animals studied included 98 yearling warmbloods fillies and colts and a convenience selected cohort of 72 resident brood mares from the larger brood mare herd (outbreak A weanling group, outbreak A brood mares), 38 mature Icelandic mares and geldings (outbreak B), and 27 horses of mixed breeds, ages and sex (outbreak C).

### 2.1 Outbreak history and management

Outbreak A occurred on a large warmblood breeding farm with a history of periodic strangles outbreaks over the previous decade. In the autumn of 2014, strangles occurred in the foals shortly after weaning.^8^ Isolation of clinically affected animals was not instituted and the outbreak was allowed to resolve over time with only supportive care. None of the foals (outbreak A, weanlings) were treated with antibiotics during clinical strangles and no resident brood mares (outbreak A, broodmares) were affected. Outbreak B occurred following the introduction of *S. equi* by 1 resident horse that had returned from a convalescence stable. As morbidity rapidly reached 100%, initial attempts to isolate affected horses were abandoned, apart from sex based pasturing and closing the farm to new introductions. Twelve horses were administered penicillin during clinical strangles. Outbreak C involved strangles introduced to a convalescent stable by a new arrival. Isolation of clinically affect animals was applied and the farm closed to new arrivals. Most horses with clinical signs of strangles were treated with antibiotics and the duration of the clinical signs for the most severely affected animals was 5 months.

### 2.2 Timing of carrier state examination

**FIGURE 1:**
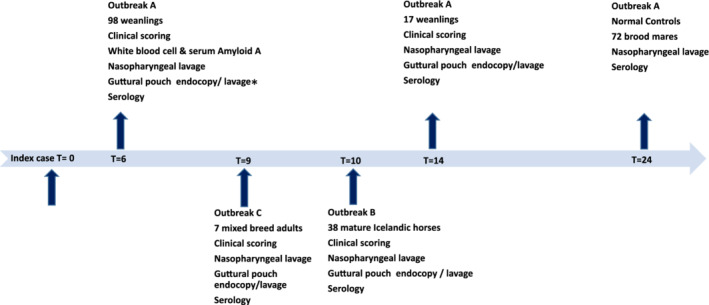
Sampling and time line for carrier detection in strangles outbreaks A, B and C, as well as control group for outbreak A. T = time in months after outbreak. *Guttural pouch endoscopy and sampling missing for one animal

As shown in Figure 1, in outbreak A 97/98 yearlings were examined 6 months after the index case and a subgroup of 17, including 15 carriers, re‐examined after a further 8 months. Additionally, 72 of the much larger population of clinically normal, but potentially exposed resident brood mares were sampled as a control subset during routine scheduled reproductive examinations approximately 24 months after the outbreak. In outbreak B, all 38 of the horses remaining on the premise were assessed for the carrier state 10 months after the index case. For outbreak C, 7/ 27 horses were examined for the carrier state 9 months after the index case.

### 2.3 Diagnostic procedures

Apart from the mares from the farm of outbreak A, all horses underwent complete clinical examinations with a uniform clinical scoring^8^ that focused on the detection of any residual abnormalities as possible consequences of strangles and included the presence of fever, defined as a rectal temperature >38.2°C, nasal discharge, lymph node swelling, or abscessation, including local scarring. Those assigning clinical variables included in the final clinical score were unaware of carrier status. Additionally, all weanlings in outbreak A, all horses in outbreak B, and 7 of 27 horses in outbreak C also underwent guttural pouch endoscopy at the time of assessment for carrier status. Visual appearance of the guttural pouches was graded by experienced endoscopists blinded to carrier status.^5^ Scoring was based on summation of grades from both guttural pouches, with 0 for no abnormalities, 1 if there was any trace of cloudy mucous in either guttural pouch, and 2 for presence of moderate to marked purulence, chondroid formation or visible remnants of scarring in the ventral aspect of the guttural pouch, providing a theoretical maximal grade of 6 (Table 1).

**TABLE 1:** Scoring scheme for clinical signs after strangles including guttural pouch endoscopy

Clinical exam	Finding		Score
Rectal temperature	≤38.2°C		0
	>38.2°C		1
Nasal discharge	None		0
	Serous		1
	Seromucoid		2
	Mucopurulent		3
	Purulent		4
Lymph node swelling	None		0
	Mild		1
	Moderate		2
	Severe		3
	Abscessation/rupture		4
Maximum score			9
Guttural pouch endoscopy	No visible abnormalities	Right pouch	0
		Left pouch	0
	Visible mucous accumulation	Right pouch	1
		Left pouch	1
	Empyema, chondroids, or mucosal defect in retropharyngeal lymph node region	Right pouch	2
		Left pouch	2
	Maximum score		6

#### 2.3.1 Detection of *S. equi*

For determination of the carrier state all horses had nasopharyngeal lavage (NPL) as per Lindahl et al,^9^ after which, with the exception of mares in outbreak A, endoscopically guided guttural pouch lavages (GPL) were performed on each guttural pouch. The guttural pouch lavages were pooled and all collected fluids centrifuged for further laboratory analysis on the subsequent working day. All lavage samples were analyzed by culture and qPCR^10^ for the presence of *S. equi*. For outbreak C, however, only qPCR positives with _ΔΔ_CT ≤ 32 were processed further for culture. Carriers were defined as any horse sampled by NPL and GPL positive to *S. equi* on at least 1 sample, either by culture or qPCR. For more information on results of diagnostic testing see Supporting Information Table 1.

#### 2.3.2 Other laboratory analysis

Whole blood was collected in potassium EDTA tubes from all foals in outbreak A and processed the same day for white blood cell counts using an automated analyzer (Sysmex KX‐21 N, Sysmex Deutschland GmbH, Norderstedt, Germany).

Serum samples were also obtained from all animals at the time of testing for carriers and stored at −20°C for subsequent analysis. All samples from the foals in outbreak A at 6 months after the index case were also analyzed for levels of serum amyloid A (SAA; immunoturbidity. Eiken Chemicals Co, Ltd, Tokyo, Japan, on automated Architect c4000 Abbott Laboratories, Abbott Park, Illinois). Finally, serum samples from all horses in outbreak A, including the mares, outbreak B, and outbreak C were analyzed for antibodies to *S. equi* using an optimized iELISA^11^ that targets both antigen A (SEQ_2190) and antigen C (SeM) of *S. equi*. Based on those workers preselected cutoff values results with optical density at 450 nm (OD_450nm_) ≥0.5 were deemed positive, ≥0.3 to <0.5 suspicious, and <0.3 seronegative.

### 2.4 Statistical methods

Descriptive statistics and, for outbreak A numerical data in outbreak A were tested for normality then compared by paired *t* test or for nonparametric data Wilcoxon ranked test.

For clinical and endoscope scoring grades contingency tables with chi square analysis was performed. The 2‐tailed Fishers exact test was used to compare carrier status to ELISA positivity to antigen A and /or antigen C. Significance was set at *P* < .05. Sensitivity, specificity, and likelihood ratios were calculated for carriers in relation to serology to *S. equi*. Finally, datasets from all 3 outbreaks were combined and logistic regression analysis performed for overall association of carrier status to clinical and serologic findings.

### 2.5 Ethical permission

This study was component of a research project on pathobiology of strangles, which was approved by the authorizing ethical committee (Uppsala Animal Ethical Committee, Diary NR C 36/14).

## 3 RESULTS

In outbreak A 15/97 were carriers at 6 months after index case, of which 3 were culture and PCR positive and 12 solely PCR positive (see Supporting Information). By 14 months after the index case examination 12/17 were carriers, of which 3 were culture positive and 9 solely PCR positive. At the 6 month evaluation the endoscopy score was significantly higher in the carriers (chi square 11.3, *P* = .003). Moreover, while there were no differences in clinical score, WBC numbers or SAA (*P* = .50‐.61). Seropositivity to *S. equi* at 6 months was significantly associated with carriers (*P* = .05). At 14 months after the index case there were no significant differences found between carriers and noncarriers for any of the indices or seropositivity (*P* = .11‐1) (Table 2). Culture positive carriers compared to noncarriers at 6 and 14 months after the index case did not differ in any of the indices, including seropositivity (data not shown). Regarding the control subset of resident mares, 3 of 72 (4%) were positive on nasopharyngeal lavage by qPCR; 1 of which was also culture positive. However, while 26 of the 72 mares (36%) were seropositive to antigen A or C, none of the carriers were seropositive, with 2/3 classified serologically as high negative, and the third, also culture positive, fully seronegative to both antigens A and C (data not shown).

**TABLE 2:** Strangles outbreak A in recently weaned warmblood horses with 53% morbidity

6 months after index (n = 97)	Noncarrier (n = 82)	Carriers (n = 15)
Clinical score	Median 2^a^ (1‐4)	Median 2^a^ (1‐3)
Endoscopy score	Median 0^b^ ^A^ (0‐3)	Median 1^b^ (0‐3)
SAA	Median 0^c^ (CI −4.3 to 47.4)	Median 0^c^ (CI −0.1 to 0.5)
WBC	10.8^d^ (CI 10.3‐11.3)	11.5^d^ (CI 9.6‐13.4)
Ag A positive	42^e^ ^B^	12^e^
A suspicious	19^B^	1
Ag C positive	4^f^ ^B^	2^f^
C suspicious	2^B^	3
Ag A or C positive	42^g^ ^B^	12^g^
A&C seronegative	17^h^ ^B^	2^h^

14 months after index (n = 17)	Noncarrier (n = 5)	All carriers (n = 12)
Clinical score	Median 0^i^ (0‐0)	Median 0^i^ (0‐2)
Endoscopy score	Median 0^j^ (0‐1)	Median 0^j^ (0‐2)
AgA positive	0^k^	4^k^
A suspicious	1	0
Ag C pos	0^l^	0^l^
C suspicious	0	0
Ag A or C positive	0^m^	4^m^
A&C seronegative	4^n^	8^n^

*Note*: Ninety‐seven animals sampled at 6 months after the index case, after which the 15 carriers detected at 6 months as well as 2 noncarriers with guttural pouch abnormalities were resampled 14 months after the index case. Figures in brackets indicate range of scores or 95% confidence intervals (CI).

Abbreviations: SAA, serum amyloid A (mg/L); WBC, white blood cell count (×10^3^/μL).

^A^ Missing 1 score.

^B^ Missing 3 analyses.

a Chi square 2.2, *P* = .53; ^b^Chi square 11.3, *P* = .003; ^c^ *P* = .61 (Wilcoxon); ^d^ *P* = .5 (*t* test); ^e^ *P* = .05; ^f^ *P* = .23; ^g^ *P* = .52; ^h^ *P* = .52 (Fishers exact test); ^i^ *P* = .2; ^j^ *P* = .79, (chi square); ^k^ *P* = .26; ^l^ *P* = 1; ^m^ *P* = .26; ^n^ *P* = 1 (Fishers exact test).

For outbreak B, 14 of 38 horses were identified as persistently infected carriers, of which 5 were culture and PCR positive and 9 solely PCR positive (see Supporting Information Table 3). Clinical score, endoscopy score and seropositivity to antigen A or C were similar for carriers versus noncarriers (*P* = .11‐1) with the majority of both carriers and noncarriers seropositive (Table 3).

**TABLE 3:** Outbreak B involving 38 mature Icelandic horses with 100% morbidity sampled 10 months after the index case

10 months after index (n = 38)	Noncarrier (n = 24)	Carriers (n = 14)
Clinical score	Median 0^a^ (0‐1)	Median 1^a^ (0‐1)
Endoscopy score	Median 0^b^ (0‐2)	Median 0^b^ (0‐2)
AgA pos	19^c^	9^c^
A suspicious	0	2
Ag C pos	13^d^	8^d^
C suspicious	5	2
Ag A or C pos	21^e^	12^e^
A&C neg	3^f^	0^f^

*Note*: Ranges in brackets.

Abbreviations: pos, positive; neg, negative.

a *P* = .24; ^b^ *P* = .35, (chi square); ^c^ *P* = .45; ^d^ *P* = 1; ^e^ *P* = 1; ^f^ *P* = .28 (Fishers exact test).

For outbreak C, there were 4 carriers identified out of the 7 horses that were tested, of which none were culture positive. Of the indices assessed between carrier and noncarriers none were significantly different. While proportionately more of the carriers were serologically positive to antigen A on the iELISA this was not significant (*P* = .5), with 1 of 3 (33%) noncarriers and 3 carriers (75%) seropositive to antigen A or C.

When data from all outbreaks were combined for analysis, findings for all carriers versus noncarriers were similar with only endoscopic score being significantly higher in carriers (chi square 22.2, *P* = .01). Moreover, while there were no significant differences for seropositivity to antigen A or C (chi square 1.4, *P* = .49, chi square 0.4, *P* = .81 respectively), significantly higher clinical scores were present in noncarriers (chi square 14.0, *P* = .02).

The sensitivity and specificity values for serology to either antigen A or C in detection of carrier varied between outbreaks and animal groups, with sensitivity ranging from 0 to 86%, specificity 47 to 100%. In turn, likelihood ratios often approached 1, but varied between 0 and infinity (Table 4).

**TABLE 4:** Relationship of carriers of *S. equi* following strangles in 3 different types of horse management systems, including comingling, to serologic status (positive OD_450nm_ ≥ 0.5, suspicious OD_450 nm_ ≥ 0.3 to <0.5, negative OD_450 nm_ < 0.3) based on % sensitivity, specificity, and resulting likelihood ratio of carrier or not

Outbreak A		Sensitivity	Specificity	Likelihood ratio positive	Likelihood ratio negative
6 months after index	Ag A pos	80 (52‐96)	47(36‐59)	1.5	0.4
	Ag Cpos	13 (2‐40)	95 (88‐99)	2.6	0.9
	Ag A or C pos	79 (49‐95)	47 (36‐58)	1.5	0.5
	Seroneg A&C	87 (60‐98)	23 (14‐33)	1.1	0.6
14 months after index	Ag A pos	33 (10‐65)	100 (48‐100)	Infinity	0.7
	Ag C pos	0 (0‐26)	100 (48‐100)	Nil	1
	Ag A or C pos	33 (10‐65)	100 (48‐100)	Infinity	0.7
	Seroneg A&C	33 (10‐65)	80 (28‐99)	1.7	0.8
Outbreak B	Ag A pos	64 (35‐87)	21 (7‐42)	0.8	1.7
	Ag C pos	57 (29‐82)	49 (26‐67)	1.1	0.9
	Ag A or C pos	86 (57‐98)	13 (3‐32)	1	1.1
	Seroneg A&C	100 (77‐100)	13 (3‐32)	1.2	0
Outbreak C	Ag A pos	75 (19‐99)	67 (9‐99)	2.3	0.4
	Ag C pos	25 (1‐81)	100 (29‐100)	Infinity	0.8
	Ag A or C pos	75 (19‐99)	67 (9‐99)	2.3	0.4
	Seroneg A&C	75 (19‐99)	33 (1‐91)	1.1	0.8

*Note*: A likelihood ratio of greater than 1 indicates the test result is associated with the disease whereas a likelihood ratio less than 1 indicates that the result is associated with absence of the disease. Tests where the likelihood ratios lie close to 1 have little practical significance. Numbers in brackets are 95% confidence intervals.

Abbreviations: neg, negative; pos, positive.

## 4 DISCUSSION

Given that animals with long‐term carriage of *S. equi* are described with the term “silent carriers” it was not surprising that clinical scoring, white blood cell counts, and serum amyloid A did not differ between carrier and noncarriers. However, as earlier reports suggested that carriers frequently are affected with guttural pouch empyema or chondroids,^12^ it was clinically relevant to identify if carriers were associated with clinical signs, or markers of systemic inflammation. While empyema or chondroids were identified in relatively few of the carriers in all 3 outbreaks, clinical scoring and inflammatory markers overlapped with noncarriers. The lack of changes to clinical or laboratory markers of inflammation suggests that the silent carrier state has negligible influence on systemic inflammatory processes.^13^

With regards to endoscopic scoring, earlier reports suggest that clear differences should be noted in many carriers.^14^ However, higher endoscope scores were only evident in the first sampling of outbreak A after 6 months. Nonetheless, even in this cohort, measureable differences were only detected in those carriers solely PCR positive. Notably, 6 of 14 carriers (3 of which were guttural pouch culture positive) were graded as 0 with no visible guttural pouch changes. Thus, on individual horses an apparently visually normal guttural pouch cannot rule out carriage of *S. equi*. Timing of guttural pouch inspection may also have been a factor in our findings, as visual differences were only observed in those animals examined in closest proximity to the initial outbreak. Earlier examinations for the carrier state^14^ detected a higher proportion of guttural pouch abnormalities, but some may have reflected a normal physiological process of general clearing of guttural pouch secretions following clinical strangles.

Incorporation of serology in the control of strangles outbreaks has shown promise through confirmation of recent exposure to *S. equi*. In particular, the iELISA utilized here has been shown to have superior sensitivity and specificity over a commercial SeM based iELISA, with the latter yielding more false positives in the presence of seroreactivity to *Streptococcus zooepidemicus* alone.^11^ Despite recent evidence to the contrary,^15^ the iELISA has also been suggested for use to detect animals that have been exposed to *S. equi* that may remain persistently infected.^2^ Accordingly,^11^ it has been proposed that horses that have recovered from strangles are also likely to generate positive iELISA test results for several months even if they have eliminated *S. equi* infection. A key potential advantage of incorporation of serology for carrier detection would be if carriers of *S. equi* maintain seropositivity longer than noncarriers. However, the outbreaks presented here clearly lacked the management of early animal separation based on serology, and thereby re‐exposure within the herds was likely.

The majority of horses in outbreaks A and all in B had seroconverted to *S. equi* by the time of testing for the carrier state.^5^ In contrast, outbreak C isolation of clinically affected animals likely prevented some resident animals from being exposed to *S. equi* during the outbreak, as evidenced by many remaining seronegative (data not shown). Thus, the proportion of animals generating a positive iELISA to *S. equi* differed between outbreaks, with proportionally fewer animals overall seropositive in association with stricter isolation routines. However, despite the iELISA discriminating horses that have been exposed to *S. equi*, some long‐term carriers in all 3 outbreaks tested seronegative at the time of carrier testing. While diversification and decay of the genome of *S. equi* has been suggested to occur in carriers,^2^, ^16^ whether such changes could explain lack of seropositivity in some carriers is yet to be determined.

The minority of carriers were identified as culture positive, which may have limited the power of our analysis. Nonetheless, serology did not readily distinguish between carrier and noncarrier in recovered groups. Moreover, although all 3 culture positive animals in outbreak A were seropositive 6 months after the outbreak, 2 of the 3 horses culture positive at 14 months were seronegative to both antigen A and C. (Supporting Information Table 2).

In addition to identifying animals that are seropositive to *S. equi* for further testing, animals with iELISA OD _450nm_ ≥ 0.3 to <0.5, deemed as “suspicious” should be retested (A. Waller, personal communication). Nonetheless, even with inclusion of those horses serologically “suspicious” there were still carriers that were fully seronegative in 2 of the outbreaks, including 2 of 14 at 6 months, 8/12 at 14 months and 1/3 in the mares in outbreak A, as well as 1/ 4 in outbreak C. Thus, adjustments to the initially determined cutoff levels for the iELISA may be in order when the intent of serology is detection of the silent carriers. While current recommendations for use of serology for carrier detection includes sampling and qPCR, this dual testing is designed to provide the security of capturing all carriers and inclusion of seropositives for further biosecurity measures. However, as no single sample from either nasopharyngeal or guttural pouch lavages effectively detects carrier state,^5^ the group defined as noncarriers in such a scheme will likely also include carriers falsely classified as negative. By definition, carriers included horses solely qPCR positive. It remains to be determined what risk such individuals pose for continued spread of strangles. Recent work has demonstrated persistence of viable bacteria in such individuals, and that such carriers can be the source of transmission of *S. equi* silently to herd‐mates.^16^ Indeed, others have indicated such animals may also pose risk of infecting naïve animals.^2^

Differing management systems along with varied biosecurity measures to limit disease spread in these 3 separate strangles outbreaks likely influenced our findings. For example, attempts to isolate and limit disease in outbreak C may have resulted in proportionally fewer seropositives at the herd level, whereas the use of antibiotics in clinical disease (outbreak B and C) could have reduced the longevity of the serological response in some individuals.^17^ As such, each strangles outbreak likely differed in the infective challenge and hence immunologic response, as shown in the wide variation in seropositivity. This also was reflected in the range of calculated sensitivities and specificities for serology to correspond to the carrier state with the highest sensitivity and specificity found in outbreak C, being similar at 75 and 67%, respectively. However, apart from selected positive likelihood ratios in outbreaks A and C, most other likelihood ratios were closer to 1 (Table 4). Thus, use of serology was of little practical significance in carrier detection since the posttest probability was little different from the pretest probability.

Of particular concern however was that 2 of the culture positive carriers 14 months after outbreak A, and the culture positive mare associated with outbreak A tested seronegative, suggesting lack of persistence of seropositivity despite carriage of viable *S. equi*.

## 5 CONCLUSIONS

Silent carriers of *S. equi* in the 3 populations of horses examined here did not appear to differ clinically or on markers of inflammation to their noncarrier herd‐mates, which may have had repeated exposure to *S. equi* being shed from carriers with which they had direct contact. Moreover, while visual changes identified by guttural pouch endoscopy may have correlated with some individuals being a carrier, many other carriers had normal appearing guttural pouches. Finally, even with isolation of affected animals or persistently infected carriers, serology alone as a surrogate marker did not effectively distinguish carriers from noncarriers. In these situations, all horses on the yard would require examination by NPL and or GPL, or both, for the presence of *S. equi* by qPCR.

## CONFLICT OF INTEREST DECLARATION

Andrew Waller through his employment at the Animal Health Trust has an economic interest in marketing the iELISA against *S. equi*. No other authors have any conflict of interest.

## OFF‐LABEL ANTIMICROBIAL DECLARATION

Authors declare no off‐label use of antimicrobials.

## INSTITUTIONAL ANIMAL CARE AND USE COMMITTEE (IACUC) OR OTHER APPROVAL DECLARATION

This study was component of a research project on pathobiology of strangles, which was approved by the Uppsala Animal Ethical Committee, Diary NR C 36/14.

## HUMAN ETHICS APPROVAL DECLARATION

Authors declare human ethics approval was not needed for this study.

## Supporting information


**Data S1**: Supporting information.
