# Supplementary material for: Markers of long term silent carriers of Streptococcus equi ssp. equi in horses
Source: J Vet Intern Med. 2020 Oct 19;34(6):2751–7. doi: 10.1111/jvim.15939 (PMC7694814; doi:10.1111/jvim.15939)
Supplement: Supplementary file 1 — Data S1: Supporting information. [file JVIM-34-2751-s001.pdf]

Supplementary Table 1. Results from 112 *S. equi* carrier positive diagnoses based on culture in comparison to ΔΔCT of PCR. Growth *S. equi* grade: 4= rich growth; 3= moderate growth; 2= scant growth; 1= single colony; 0 = no growth (± SD = standard deviation).

**Growth *S. equi* Grade**	**Mean ΔΔ CT PCR (± SD)**
4 (n=22)	23.2 (± 3.2)
3 (n=11)	27.8 (± 3.5)
2 (n=12)	30.6 (± 3.7)
1 (n=11)	29.6 (± 2.4)
0 (n=56)	33.9 (± 3.0)

Supplementary Table 2: Serology in *S. equi* carriers culture-positive versus solely PCR-positive from strangles outbreak A in recently weaned warmblood horses. Ninety-seven animals were sampled at 6 months post index case, after which the 15 carriers detected at 6 months as well as two non-carriers with guttural pouch abnormalities were resampled 14 months after the index case.

**Carriers 6months post index 15/97**	**Culture positive n=3**	**Solely qPCR positive n=12**
Ag A positive	3	9
A suspicious	0	1
Ag C positive	1	1
C suspicious	0	3
Ag A or C positive	3	9
A&C seronegative	0	2
**Carriers 14 months post index 12/17**	**n=3**	**n=9**
AgA positive	1	3

A suspicious	0	0
Ag C positive	0	0
C suspicious	0	0
Ag A or C positive	1	3
A&C seronegative	2	6

Supplementary Table 3. Serology in *S. equi* carriers culture-positive versus solely PCR-positive in outbreak B involving 38 mature Icelandic horses with 100 % morbidity sampled 10 months after the index case.

**Carriers after 10 months 14/38**	**Culture positive n= 5**	**Solely qPCR positive n=9**
**AgA positive**	2	7
**A suspicious**	1	1
**Ag C positive**	3	5
**C suspicious**	1	1
**Ag A or C positive**	4	8
**A&C negative**	0	0
